# Click-Chemistry Based High Throughput Screening Platform for Modulators of Ras Palmitoylation

**DOI:** 10.1038/srep41147

**Published:** 2017-01-23

**Authors:** Lakshmi Ganesan, Peyton Shieh, Carolyn R. Bertozzi, Ilya Levental

**Affiliations:** 1 Department of Integrated Biology and Pharmacology, McGovern Medical School, University of Texas Health Science Center at Houston, Houston, TX 77030, USA; 2 Department of Chemistry, Stanford University, Stanford, CA 94305, USA; 3 Howard Hughes Medical Institute, Stanford University, Stanford, CA 94305, USA

## Abstract

Palmitoylation is a widespread, reversible lipid modification that has been implicated in regulating a variety of cellular processes. Approximately one thousand proteins are annotated as being palmitoylated, and for some of these, including several oncogenes of the Ras and Src families, palmitoylation is indispensable for protein function. Despite this wealth of disease-relevant targets, there are currently few effective pharmacological tools to interfere with protein palmitoylation. One reason for this lack of development is the dearth of assays to efficiently screen for small molecular inhibitors of palmitoylation. To address this shortcoming, we have developed a robust, high-throughput compatible, click chemistry-based approach to identify small molecules that interfere with the palmitoylation of Ras, a high value therapeutic target that is mutated in up to a third of human cancers. This assay design shows excellent performance in 384-well format and is sensitive to known, non-specific palmitoylation inhibitors. Further, we demonstrate an ideal counter-screening strategy, which relies on a target peptide from an unrelated protein, the Src-family kinase Fyn. The screening approach described here provides an integrated platform to identify specific modulators of palmitoylated proteins, demonstrated here for Ras and Fyn, but potentially applicable to pharmaceutical targets involved in a variety of human diseases.

Protein palmitoylation is a reversible post-translational regulator of hundreds, if not thousands, of proteins^1^. For many of these proteins, palmitoylation serves a crucial regulatory role that is facilitated by the reversibility of this modification^2^, which stands in contrast to all other protein lipidations, which are irreversible. There are three variations of protein palmitoylation (S-, N- and O-palmitoylation), with S-palmitoylation by far the most abundant and well-studied. S-palmitoylation modifies both peripheral and integral membrane proteins, and is carried out by a family of CRD (cysteine-rich domain)-containing palmitoyl acyl transferases (PATs)^3^, which possess the characteristic Asp-His-His-Cys (DHHC) motif, and have overlapping specificities^3^. Less is known about the de-palmitoylating enzymes (otherwise known as acyl-protein thioesterases) though the list of enzymes with this activity has expanded recently from only three (APT1/2^4^ and PPT1^5^) to potentially many more^6^. S-Palmitoylation often, although not always, occurs as a second lipid modification, and serves to confer stable membrane anchorage to proteins that transiently interact with the membrane through myristoyl/prenyl groups. For several proteins, including but not limited to, most members of the Ras family of GTPases^7^ and several Src-family kinases (including Fyn, Lck, and Lyn)^8^, S-palmitoylation is indispensable for membrane localization and subsequent signaling^9^. Recent advances in chemical biology based on biorthogonal click chemistry have expanded and elucidated many novel cellular targets and functions of S-palmitoylation^10^,^11^.

Despite its ubiquity and biomedical relevance, there are few chemical tools available for the perturbation of S-palmitoylation, and none have been pursued for clinical translation. The most commonly used reagent for inhibition of palmitoylation is the non-specific palmitate analog 2-Bromopalmitate (2BP), which covalently modifies the active site of DHHC PATs *in vitro*^12^ as a ‘suicide inhibitor’. The drawbacks of this reagent are that it is very hydrophobic, likely has low bioavailability, and most importantly, is extremely promiscuous with respect to targets^13^,^14^, modifying not only PAT enzymes but also palmitoylation targets and a variety of other proteins. Other non-specific PAT inhibitors (including 2-(2-hydroxy-5-nitro-benzylidine)-benzothiophen-3-one, cerulenin, and tunicamycin) have been reported^12^,^15^,^16^, although none of these have shown the efficacy or specificity sufficient to motivate further investigation of clinical utility. The dearth of effective pharmacological tools for probing protein palmitoylation is due in large part to a lack of robust, screening-compatible assays. To address this shortcoming, we have developed a high-throughput compatible *in vitro* assay to identify specific inhibitors of protein S-palmitoylation.

As a therapeutically-relevant target, we focused on the oncogene Ras, a small GTPase that acts as a key switch in a number of cell signaling pathways that regulate cell growth, survival, proliferation, and differentiation^17^,^18^. Consistent with this crucial role in regulating mitogenesis, Ras mutations are sufficient for oncogenic transformation and associated with 20–30% of all human cancers^19^. Even in cancers lacking Ras mutations, there is often significant hyper-activation of Ras-regulated signaling pathways, due to exaggerated growth factor-mediated signaling^20^. However, despite decades of research, Ras has proven intransigent to pharmacological intervention, temporarily earning the unfortunate moniker of ‘undruggable target’ due to its high affinity for GTP and the lack of clear allosteric binding pockets^21^.

Ras interacts both with upstream regulators and downstream effectors at the plasma membrane, making membrane anchoring indispensable for Ras-mediated signaling^20^,^22^, and suggesting that inhibition of this anchoring could be a viable therapeutic strategy^23^. All four known Ras proteins (N-Ras, H-Ras, and the splice-variants K-Ras4A and K-Ras4B) interact transiently with the membrane via a C-terminal isoprenyl group. Prenylation inhibitors generated significant enthusiasm, but were clinically unsuccessful due to untenable toxicity associated with other prenylated cellular proteins^24^. For N-, H-, and K-Ras4A, stable membrane anchoring requires the post-translational addition of palmitic acid residues via S-acylation of intracellular cysteines (S-palmitoylation). Critically, this ‘palmitoylation’ is essential for Ras oncogenic signaling^25^, suggesting its inhibition as an intriguing strategy for interference with Ras-associated oncogenesis. Importantly, palmitoylation is dynamic and reversible, implying a regulatory role in cell signaling. Moreover, unlike prenylation, palmitoylation is mediated by a variety of different enzymes^26^. So far, only one of the 23 known PATs has been associated with Ras palmitoylation - the *zDHHC9-GCP16* complex^3^,^26^,^27^,^28^. Our target-based approach (Fig. 1) uses a truncated synthetic peptide comprised of the minimal membrane-anchoring region of the N-Ras isoform (Fyn for the counter-screen), which contains the native palmitoylation site of N-Ras (on Cys181), and is palmitoylated *in vivo*^29^. The peptides retain the covalently linked primary lipid modifications (C-terminal farnesyl and N-terminal myristoyl groups, of fully processed N-Ras and Fyn, respectively) to ensure specificity. We demonstrate that these peptides are enzymatically palmitoylated *in vitro* and that known palmitoylation inhibitors can be effectively detected in 384-well format. Finally, we demonstrate a robust counter-screening strategy that constitutes a comprehensive platform for discovery and development of palmitoylation-targeted pharmaceuticals.

## Results

**Figure 1:**
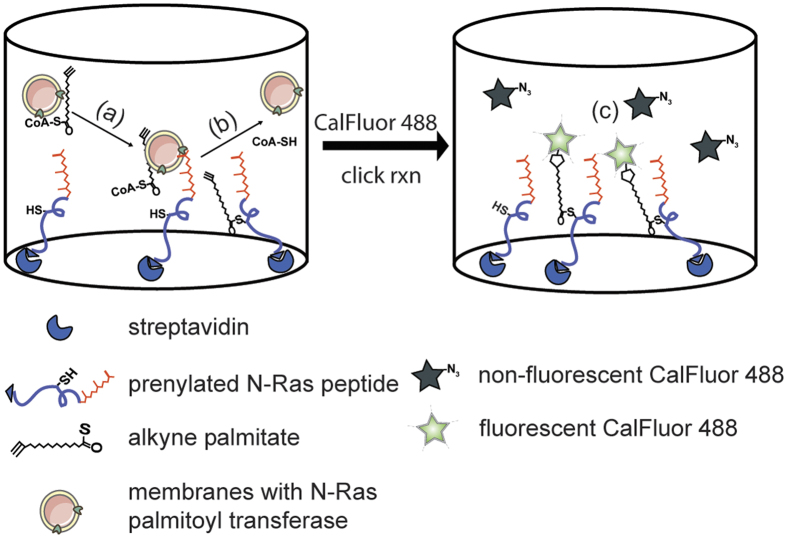
Schematic representation of the screening assay. (**a**) N-terminal biotinylated, C-terminal farnesylated N-Ras peptide was captured on streptavidin-coated plates. (**b**) Palmitoylation of N-Ras was initiated by the addition of alk–palm–CoA in the presence of a membrane preparation from MDCK cells. (**c**) The unreacted alk–palm–CoA was removed, followed by 1,3-dipolar cycloaddition with fluorogenic CalFluor 488, which is weakly fluorescent but becomes bright upon click reaction.

We have developed a robust, high-throughput compatible assay platform to identify inhibitors of palmitoylation (Fig. 1). Below we describe the development and characterization of this assay in 384-well format, in addition to demonstrating a counter-screening strategy that ensures specificity of identified hit compounds.

### Peptide binding capacity

**Figure 2:**
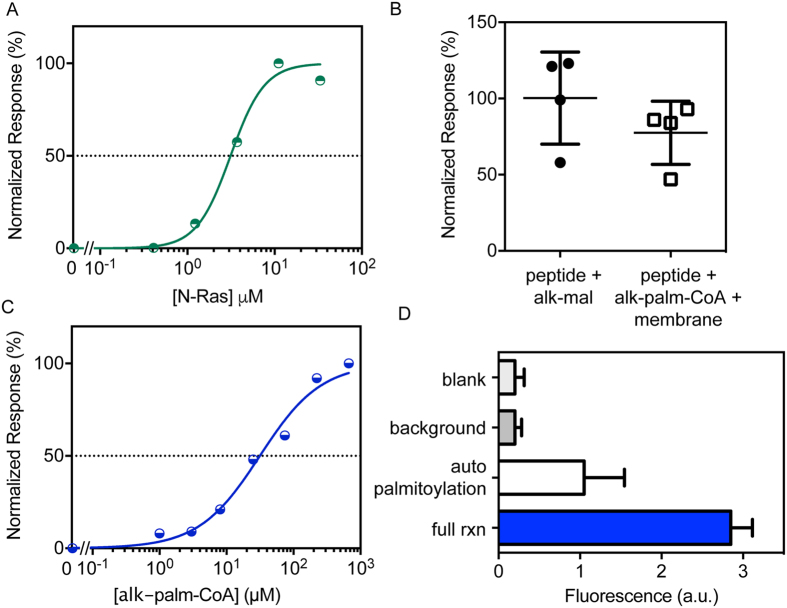
Assay design and characterization. (**A**) A click-enabled, cysteine-reactive probe (alk–maleimide) was used to measure saturation binding of biotinylated N-Ras peptide on streptavidin-coated plates. From this analysis 10 μM peptide was chosen as the near-saturating concentration for coating plates. (**B**) Palmitoylation of N-Ras peptide using the biorthogonal probe alk–palm–CoA in the presence of MDCK membranes was nearly as efficient (~80%) as labeling with alk–maleimide. (**C**) Dose-response curve was obtained using various concentrations of alk–palm–CoA to determine the linear range of the assay. (**D**) Typical controls included in the assay in the 384-well format are blank (click reagents only; no peptide or membrane), background (no target peptide), auto-palmitoylation (no membrane) and palmitoylation (full rxn). Data shown is the average ± SD for n = 3 independent experiments.

A binding curve of the biotinylated N-Ras peptide to the streptavidin-coated plate was constructed using a cysteine-reactive, click-enabled probe (maleimide–PEG_4_–alkyne) (Fig. 2A). This curve shows saturation because of the limited availability of binding sites for biotinylated peptide. Based on this analysis, a concentration of 10 μM N-Ras peptide, a near-saturating concentration in the linear range of detection, was selected for coating the streptavidin plates for the palmitoylation experiments.

### N-Ras palmitoylation detection

The native palmitoyl transferase (PAT) for Ras is a hetero-oligomer of two large, multi-pass membrane proteins (the catalytic DHHC9 and co-enzyme GCP16)^27^, presenting a significant hurdle for biochemical purification. Additionally, the 23 mammalian PATs show significant redundancy and substrate overlap, suggesting that inhibition of solely DHHC9/GCP may not efficaciously inhibit all Ras palmitoylation. Together, these factors recommend the target-based approach (rather than enzyme-based), which has recently been successful in identifying a target-specific N-acylation inhibitor^30^. Here, the enzyme is not purified, but rather included in a cellular membrane preparation from cells with known Ras-palmitoylation activity^31^, as previously demonstrated^32^. We confirmed that this preparation contains the necessary machinery for *in vitro* Ras palmitoylation-depalmitoylation activity by a novel, click chemistry-based fluorogenic palmitoylation detection scheme (Fig. 2B). Using the biorthogonal probe ω-alkyne palmitoyl coenzyme A (alk–palm–CoA), we were able to detect and quantify N-Ras palmitoylation using a novel fluorogenic probe CalFluor 488^33^,^34^, which is essentially non-fluorescent until it participates in 1, 3-dipolar cycloaddition (click) reaction with an alkyne-containing moiety (Fig. S1). Thus, in our assay, background-subtracted fluorescent signal is the direct result of, and is stoichiometric to, the levels of N-Ras palmitoylation.

**Figure 3:**
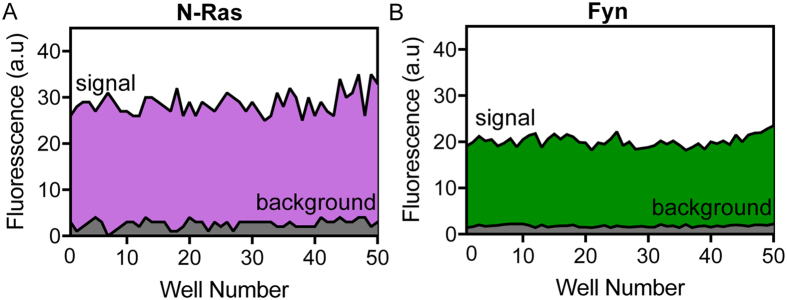
Evaluating assay performance. Blank subtracted signals and background for palmitoylation reaction with (**A**) N-Ras and (**B**) Fyn target peptides for 50 wells of a 384-well plate, used to calculate assay statistical parameters (see Table 1).

The palmitoylation reaction carried out using the captured N-Ras peptide and alk–palm–CoA produced fluorescent signals ~80% of the cysteine reactive probe maleimide–PEG_4_–alkyne, used here as the maximum possible signal for a fixed concentration of bound peptide, due to its exhaustive reactivity with free cysteines (Fig. 2B). A dose-response relationship was established using increasing concentrations of alk–palm–CoA (Fig. 2C) and 15 μM was chosen for performing the palmitoylation assay. Figure 2D depicts a typical control experiment in 384-well format. Wells without peptide (background) show no fluorescent signal above blank (only click reagents; no peptide or membrane). Without addition of membrane to catalyze the reaction, there is a notable increase in fluorescent signal, likely indicative of non-enzymatic auto-palmitoylation. This signal is enhanced by >2-fold by the membrane preparation that contains the enzymatic machinery for N-Ras palmitoylation. The assay showed an excellent separation between sample and background signals, yielding a *Z’* score of 0.62 with the N-Ras peptide (Fig. 3A). Moreover, the assay was insensitive to DMSO up to 3% (Fig. S2).

### Counter-screening with Fyn peptide

The assay described above is highly robust for the case of the Ras peptide. To demonstrate the modularity of the assay design and its transferability to other targets, while simultaneously demonstrating an ideal counter-screening strategy, we replaced the N-Ras peptide with an unrelated target peptide containing the minimal membrane anchoring sequence (including the myristoylated Gly 2) and palmitoylation sites (Cys 3 and 6) of the Src-family kinase Fyn. Using identical conditions to those established for N-Ras, the screen with Fyn also showed excellent separation between sample and background signals (Fig. 3B) with a *Z’* score 0.8. The comparisons between Ras and Fyn are summarized in Table 1, and show that the assay performs similarly well in both conditions, and is highly reproducible with respect to between-plate and day-to-day variations. The slight differences in assay performance between the two peptides may be due to the presence of two palmitoylation sites (Cys 3 and 6) on Fyn compared to a single site on N-Ras (Cys 181), differential efficiencies of palmitoylation, or different rates of palmitate turnover.

### Dose-dependent responses to palmitoylation inhibitors

**Figure 4:**
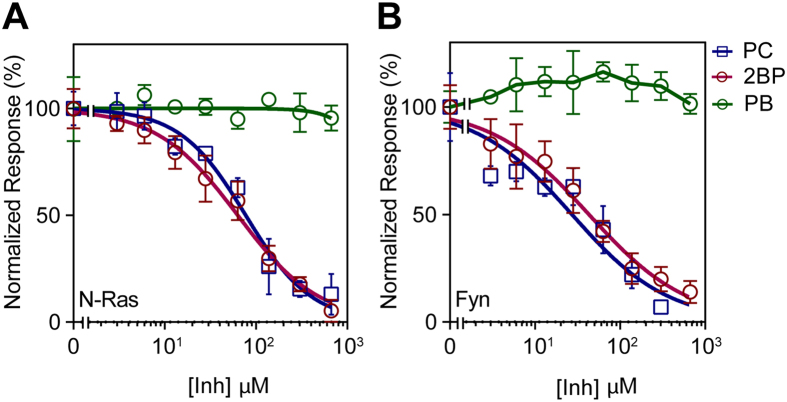
Evaluation of known modulators of protein palmitoylation. Concentration-dependent inhibition curves for (**A**) N-Ras and (**B**) Fyn palmitoylation by unlabeled palmitoyl CoA (PC), 2-bromopalmitate (2BP), and Palmostatin B (PB). Data shown are average ± SD for n = 3 independent experiments.

To further validate the assay, we determined the dose responses to known modulators of palmitoylation: 2-bromopalmitate (2BP)^35^, palmostatin B^4^, and palmitoyl-CoA (PC). 2BP is a commonly used covalent inhibitor of palmitoylation that promiscuously reacts with over 450 targets that include both palmitoylated and non-palmitoylated proteins^14^. Palmostatin B is an inhibitor of the most widely characterized palmitoyl thioesterase (i.e. depalmitoylase) APT1. Native (i.e. not alkyne) PC is a direct competitive inhibitor for our assay, by displacing alk–palm–CoA from the enzymes and occupying available palmitoylation sites on the peptide. Both putative inhibitors (2BP and PC) showed clear dose-dependent inhibition of the palmitoylation reaction for both the N-Ras and Fyn peptides (Fig. 4). The concentration at half-maximal inhibition (*IC*_*50*_) of both inhibitors were very similar for the two peptides, confirming their non-specific mechanisms of action (Fig. 4). The *IC*_*50*_ values (77 and 27 μM for N-Ras and Fyn, respectively) for the PC were in line with expectation, i.e. approximately equimolar with alk–palm–CoA. For 2BP, the values were 63 and 43 μM (N-Ras and Fyn), in very good agreement with the potencies previously demonstrated with purified enzymes^12^,^36^. Neither compound had any appreciable effect when it was pre-incubated with peptide rather than the membrane (Fig. S3), suggesting that the mechanism of action was inhibition of the palmitoylation enzymes in the membrane preparation, rather than direct reaction with the target peptides. The APT1 inhibitor Palmostatin B (PB) had no effect on palmitoylation of N-Ras, and slightly enhanced the palmitoylation of Fyn, as expected. The relatively high levels of palmitoylation observed in the assay (i.e. ~80% of all cysteines are palmitoylated – see Fig. 2B) and lack of major effect of the ‘depalmitoylase’ inhibitor (PB) both suggest that the palmitoylation reaction is dominant in our assay. This may be because the depalmitoylation enzymes are peripheral membrane proteins that are depleted from the membrane preparations, or because hydrolase inhibitors in the protease inhibitor cocktail used during membrane preparation irreversibly inactivate the depalmitoylases.

### Compatibility for high-throughput screening

**Figure 5:**
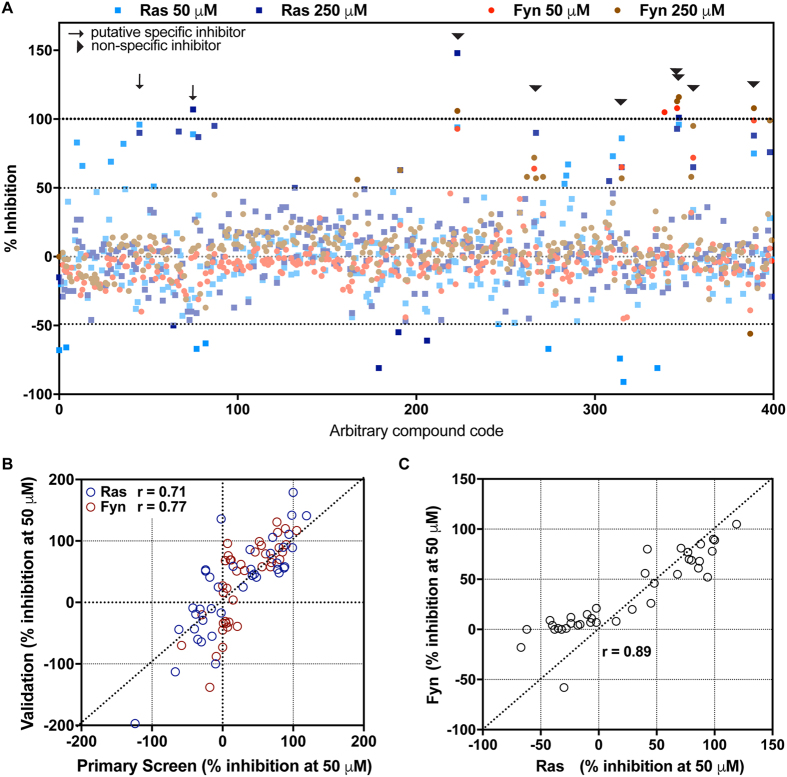
Demonstration of high-throughput screening capability. (**A**) 400 compounds from the NCI Diversity Set library were screened at two concentrations (50 and 250 μM) against both N-Ras (blue) and Fyn (red) target peptides. ~95% of compounds did not achieve an arbitrary cutoff of 50% inhibition/promotion of palmitoylation. Highlighted in arrowheads are compounds that showed activity at both doses, but without notable specificity for N-Ras or Fyn. Highlighted with arrows are compounds with activity at both doses and specificity for N-Ras over Fyn. (**B**) The reproducibility of the screening assay in 384-well format was tested by re-evaluating a range of compounds from the primary screen in small-scale validation runs. There were good correlations for both peptides at 50 μM between the primary and validation datasets. (**C**) Most of the identified compounds were not specific for either peptide, suggesting that they are PAINS and interfere with the assay rather than specific palmitoylation.

We used a subset of 400 compounds from the National Cancer Institute’s (NCI) Diversity Set V for a pilot study to (i) evaluate the scalability of the screening assay to a 384-well format and (ii) assess its ability to identify modulators of N-Ras palmitoylation and (iii) test the ability of the counter-screen to identify modulators that show preferential activity towards either of these two valuable therapeutic targets. Figure 5 shows the results from manual screening of these 400 compounds at two concentrations (50 and 250 μM) against both Fyn and N-Ras target peptides over six 384-well plates. On each plate, dose response curves of known inhibitors palm-CoA and 2-BP were included as positive controls, yielding data similar to Fig. 4 (not shown). Percent inhibition was calculated using background (no peptide) - subtracted fluorescent signals from test and control wells:


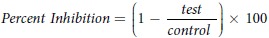


Where “test” denotes signal from wells with added test compounds and “control” denotes averaged signal from wells with no inhibitor.

An arbitrary cutoff of 50% inhibition or promotion was applied to identify compounds that robustly affected palmitoylation of the peptides, with ~95% failing to achieve that cutoff, as expected from an untargeted library screen (Fig. 5A). Furthermore, no compounds were observed to promote palmitoylation (% inhibition <−50%) of either N-Ras or Fyn at both tested concentrations. However, several compounds from this limited screen showed potent inhibition of palmitoylation. The results from the primary screen in 384-well format were highly reproducible, as demonstrated by strong correlations between the same compounds assayed in small-scale validation experiments (Fig. 5B). Most of the hits were non-specific (arrowheads in Fig. 5A), reducing signal in both N-Ras and Fyn test wells. These compounds could be broad-spectrum palmitoylation inhibitors, but more likely are compounds that interfere non-specifically with the assay. The most likely non-specific hits are so-called Pan-Assay Interference Compounds (PAINS)^37^, specifically detergent-like molecules that disrupt the membranes or directly cysteine-reactive agents. This is demonstrated by the strong correlation between inhibitory activities against both peptides in a subset of compounds chosen from the primary screen to span a range of inhibitor efficiencies (Fig. 5C). However, two compounds showed specific activity, highlighted by the arrows in Fig. 5A. These will be evaluated in future work to determine their activity in cell-based assays.

## Discussion

Although protein palmitoylation is a post-translational regulator of hundreds, if not thousands, of signaling proteins^1^, there are few chemical tools available for its perturbation, and none have been pursued for clinical translation. To address this shortcoming, we have developed a high throughput-compatible *in vitro* assay for identifying inhibitors of S-palmitoylation of specific proteins. Our target-based approach uses a truncated synthetic peptide comprised of the minimal membrane-anchoring region of the N-Ras isoform. The counter-screening strategy consists of a peptide based on an alternative palmitoylated protein, Fyn, which is also palmitoylated, but by a different enzyme^3^,^38^. The palmitoylation machinery was introduced into the assay via membrane extracts from cells with known N-Ras-palmitoylating activity^39^. This strategy was designed to serve two purposes – (i) to preserve the multipass transmembrane proteins’ native structure and function by keeping them in their biological context; (ii) the known promiscuity of DHHC PATs^3^ recommends a target-focused, rather than enzyme-focused, approach.

The therapeutic potential for targeting Ras‒membrane association was recently reviewed, concluding that this approach was “both logical and potentially tractable”^40^. For most of the Ras isoforms, palmitoylation is required for stable membrane association, as has been shown by a number of groups in high profile papers over the last decade^31^,^39^,^41^. Most importantly, this requirement for palmitoylation-induced membrane association is essential for Ras-driven oncogenesis^25^,^42^. Our approach is specifically designed to target the palmitoylated isoforms of Ras, namely N-Ras, H-Ras, and K-Ras4A - a splice-variant of K-Ras4B. KRAS (the gene for both K-Ras isoforms) is the variant most commonly associated with cancer (~30% of all human tumors), although N-Ras is also frequently mutated and oncogenic (~15% of all Ras-driven tumors), while H-Ras is less common^18^. Importantly, K-Ras4A (the palmitoylated variant) was considered a minor, tissue-specific variant until a recent report demonstrated that it is indeed widely and robustly expressed, and its mutation is sufficient for tumorigenesis^43^. Thus, many cancers ascribed to the non-palmitoylated K-Ras4B isoform may be susceptible to inhibition of Ras palmitoylation.

Although prenylation (farnesylation and geranylation) and palmitoylation are similar in that they are both lipid modifications, they are different in many other respects. The two most important are *reversibility* and *enzyme multiplicity*. Prenylation is co-translational and required for stability of all Ras variants, among many other proteins. In contrast, palmitoylation is a post-translational, regulatory process that is not essential for protein stability, but often crucial for localization and activity. Moreover, while there are only 3 enzymes that mediate prenylation (and these are largely cross-redundant), there are 23 palmitoyl transferases that have less overlapping substrate specificities^3^,^44^. Consistent with the multiplicity of enzymes mediating palmitoylation, there is no single consensus motif to indicate a palmitoylation site^45^,^46^, in contrast to the CAAX motif that specifies a prenylation site. Thus, while inhibiting prenylation is inherently non-specific, specificity is more likely to be accessible for palmitoylation.

Previous attempts to inhibit Ras activity via interference with prenylation were quite successful, with potential drugs proceeding as far as human clinical trials. Unfortunately, these compounds ultimately failed in the clinic for two major reasons: (a) the prenylation enzymes are largely functionally redundant; (b) there are many prenylated proteins but few prenylating enzymes – thus broad inhibition of the prenylation machinery is achievable but untenably toxic due to off-target effects^40^,^47^. We propose that targeting Ras palmitoylation will be less subject to these issues because the enzymes for palmitoylation are widely divergent and substrate specific^3^,^38^, and our target-based approach is designed to identify inhibitors of the process rather than a specific enzyme.

## Conclusion

Protein palmitoylation is a ubiquitous post-translational modification and is indispensable for the localization and biological function of many therapeutically relevant targets; however, no specific pharmacological inhibitors are yet available. Here, we demonstrate a high-throughput compatible assay capable of identifying specific inhibitors of palmitoylation of target peptides. As proof-of-principle, we have focused on N-Ras, a member of the Ras family of oncogenes which is frequently mutated in human cancers, yet remains elusive to pharmacological intervention. Our demonstration of a successful assay for screening specific protein palmitoylation inhibitors opens the door to inhibiting palmitoylation in proteins responsible for a variety of human diseases, including hyper/autoimmunity (e.g. Lck, CD4, CD8), cardiovascular disease (phospholemman), neurodegenerative disease (Huntingtin), viral infection (influenza hemagglutinin), and a large subset of cancers (estrogen receptor, Ras, Fyn).

## Materials and Methods

### Materials

Pierce™ Streptavidin-coated, high capacity, black 384-well plates were obtained from Life Technologies (Grand Island, NY), alkyne-palmitoyl-CoA was obtained from Cayman Chemicals (Ann Arbor, MI), MDCK (Madin-Darby canine kidney epithelial) cells (ATCC^®^ CCL-34™) were obtained from ATCC (Manassas, VA), the fluorogenic probe CalFluor 488 was synthesized by the Bertozzi lab; palmitoyl CoA, 2-bromopalmitate, 2-(N–morpholino) ethanesulfonic acid (MES), and tris(2-carboxyethyl)phosphine (TCEP) were obtained from Sigma Aldrich (St. Louis, MO); 3 [tris (3-hydroxypropyltriazolylmethyl) amine (THPTA), maleimide–PEG_4_–alkyne, and biotin–PEG_4_–alkyne were obtained from Bioconjugate Technologies (Scottsdale, AZ).

### N-Ras Peptide

A short synthetic N-Ras peptide *(Biotin* – *(PEG)*_*3*_– *LNSSDDGTQGC (*–*SH) MGLP (C*–*Farnesyl)* – *OMe)* that comprises the minimal membrane-anchoring region of N-Ras isoform, containing the free native palmitoylation site (Cys 181) was custom synthesized by Phoenix Pharmaceuticals (Burlingame, CA). To ensure specificity, this peptide retained the covalently linked farnesyl group and C-terminal O-methylation of native, fully processed N-Ras, along with an N-terminal biotin tag, separated by a PEG_3_-spacer arm for capture using streptavidin coated 384-well plates.

### Fyn-peptide

For the counter-screen, the N-Ras peptide was replaced with that of the minimal membrane anchoring region of Fyn *(G (N*–*Myristoyl) C (*–*SH) VQC (*–*SH) KDKEATKLTE (PEG)*_*3*_–*Biotin),* custom synthesized by Pierce Biotechnology (Rockford, IL). The synthetic Fyn peptide contained the native palmitoylation sites (Cys 3 and 6)^48^, an N-terminal myristoyl group attached to Gly 2, and a C-terminal biotin tag separated by a PEG_3_-spacer arm.

### Peptide Saturation experiments

To determine the amount of peptide to be used in the palmitoylation assay, a dose-response titration using indicated concentrations of peptide diluted in PB (*Palmitoylation Buffer (PB) -* 100 mM MES, 0.5 mM TCEP, *p*H 7.2) was performed (Fig. 2A). Equal volumes (20 μL) of peptide and 1 mM maleimide–PEG_4_–alkyne, were allowed to react in a BSA-blocked plate with intermittent mixing at 300 rpm for 1 hr at 23 °C. The unreacted maleimide was quenched with 10-fold molar excess of free cysteine for 1 hr at 23 °C. The above mixture was transferred to a streptavidin-coated plate to capture the peptide — maleimide–PEG_4_ –alkyne conjugate. After 1 hr of capture, the plates were washed using CB (*Click/Wash Buffer (CB)* **-** 100 mM MES, 0.2% v/v Triton X, *p*H 7.2) and the captured peptide was detected and quantified by 1–3, dipolar azide-alkyne cycloaddition (click) reaction described below.

### High Throughput Screening Assay for Modulators of Protein Palmitoylation

#### Step 1: Peptide Capture

The biotinylated peptide was dissolved in PB to a final concentration of 10 μM. It was captured in a streptavidin-coated 384-well plate (10 μL/well) by incubation for 1 hr at 37 °C.

#### Step 2: Pre-incubation of native membrane preparation with the test compounds

The machinery necessary for enzymatic palmitoylation was added to the assay as a P_60_ membrane preparation from cells possessing Ras-palmitoylation activities, namely MDCK cells. The cells were cultured to 90% confluency in in Dulbecco’s Modified Eagle Medium (DMEM) supplemented with 10% fetal bovine serum (FBS), 100 U/mL penicillin and 100 μg/mL streptomycin. They were lysed by sonication into hypotonic buffer (50 mM Tris base pH 7.4) with added protease inhibitor cocktail (1 mM Pefabloc SC, 0.8 μM Aprotinin, 50 μM Bestatin, 15 μM E-64, 20 μM Leupeptin, and 10 μM Pepstatin). They were centrifuged at 500 g, 5 min to remove cellular debris. The supernatant was transferred to fresh eppendorf tubes and centrifuged at 60,000 rpm, 1 hr 30 min, at 4 °C to obtain the whole (P_60_) membrane preparation. This preparation was snap-frozen under liquid nitrogen and stored at −80 °C for use in the assay as a source of enzyme activity. At the time of performing the assay, the membrane pellet was dissolved in PB (10 μL, ~0.5 mg of total protein/ml) and simultaneously pre-incubated for 1 hr at 37 °C with 10 μL of compound/vehicle, also diluted in PB.

#### Step 3: Palmitoylation reaction

The pre-incubated membrane-compounds mix was added to the immobilized peptide (10 μL/well), followed by 10 μL/well of 3X ω-alkyne palmitoyl coenzyme A (final concentration of 15 μM) to initiate the palmitoylation reaction. The mixture was incubated for 30 min at 37 °C to allow the palmitoylation reaction to proceed to completion. The wells were washed thrice with CB (40 μL/well) to remove all of the membrane extract and any unreacted *ω*-alkyne palmitoyl CoA.

#### Step 4: Alkyne-Azide cycloaddition (click reaction)

The click reaction mix was prepared by adding to CB the following reagents (final concentrations and order of addition specified below): CalFluor 488 (45 μM), mixed copper sulfate (0.1 mM) and THPTA (0.5 mM), then sodium ascorbate (10 mM). The click reaction mix was added to the wells (30 μL/well) and the click reaction was carried out for 30 min at 37 °C with vigorous mixing. The wells were washed three times with CB (40 μL/well) to remove unreacted azide. The use of a fluorogenic probe increased signal/background by ~14-fold compared to a simple clickable fluorescent probe (azido-Alexa488) (Fig. S4).

#### Step 5: Detection

The fluorescent alkyne-azide cyclo-adduct was detected using TECAN Infinite M200 multi-plate fluorescent reader with excitation/emission wavelengths at 488/520 nm. The schematic for the assay as described above is depicted in Fig. 1.

### Data fitting and statistics

All dose-response experiments were done in duplicate or triplicate per plate and repeated at least twice. The averaged data was normalized and used for data fitting and analysis. Saturation binding experiments were fit using the standard log agonist vs. response model 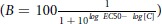. Competitive dose-response curves were fit using the standard log inhibitor vs. response model 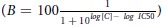. All dose-response curve fittings were done using GraphPad Prism 6.0 (La Jolla, CA).

## Additional Information

**How to cite this article**: Ganesan, L. *et al*. Click-Chemistry Based High Throughput Screening Platform for Modulators of Ras Palmitoylation. *Sci. Rep.* **7**, 41147; doi: 10.1038/srep41147 (2017).

**Publisher's note:** Springer Nature remains neutral with regard to jurisdictional claims in published maps and institutional affiliations.

## Supplementary Material

Supplementary Figures

## Figures and Tables

**Table 1:** Assay Statistical Parameters.

	S	Σ_s_	CV	S/B	Z'	n
N-Ras	28.7	2.4	0.2	10.9	0.6	50
Fyn	20.2	1.2	0.1	11.4	0.8	50

Summary of statistical parameters: S – average signal; σ_s_ – standard deviation; CV – coefficient of variation; S/B – Signal-to-background ratio; Z’ – Z’ score as determined by 
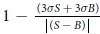
; n – number of samples.
