# Supplementary material for: Click-Chemistry Based High Throughput Screening Platform for Modulators of Ras Palmitoylation
Source: Sci Rep. 2017 Jan 23;7:41147. doi: 10.1038/srep41147 (PMC5255568; doi:10.1038/srep41147)
Supplement: Supplementary Figures [file srep41147-s1.pdf]

Title: Click-Chemistry Based High Throughput Screening Platform for Modulators of Ras Palmitoylation

Authors: Lakshmi Ganesan[1], Peyton Shieh[2], Carolyn R. Bertozzi[2, 3], Ilya Levental*[1]

[1] Department of Integrated Biology and Pharmacology, McGovern Medical School, University of Texas at Houston Health Science Center, Houston, TX 77030, USA

[2] Department of Chemistry, Stanford University, Stanford, CA 94305, USA

[3] Howard Hughes Medical Institute, Stanford University, Stanford, CA 94305, USA

*Corresponding author

Ilya Levental (ilya.levental@uth.tmc.edu)

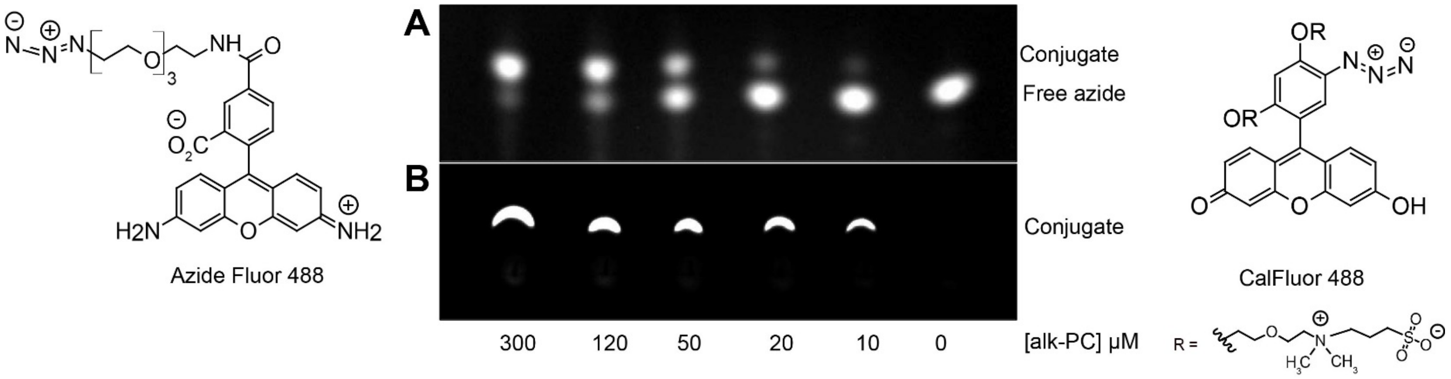

Supplementary Figure S1: Enhanced signal/noise using a fluorogenic probe. Copper (I)-catalyzed, 1,3 –dipolar cycloaddition reaction was carried out in the presence of indicated concentrations of alk–palm-CoA using a fluorescent probe Azide 488 (A) and the fluorogenic probe, CalFluor 488(B). The reaction mixture was spotted on a silica-gel coated TLC plate, developed using n-butanol: water: acetic acid (5:3:2) as mobile phase and visualized using ChemiDoc™ MP Imaging system (BioRad) with Ex/Em set to 488/520 nm. The fluorogenic probe leads to dramatic enhancement of signal / background in the plate assay (see Fig S4).

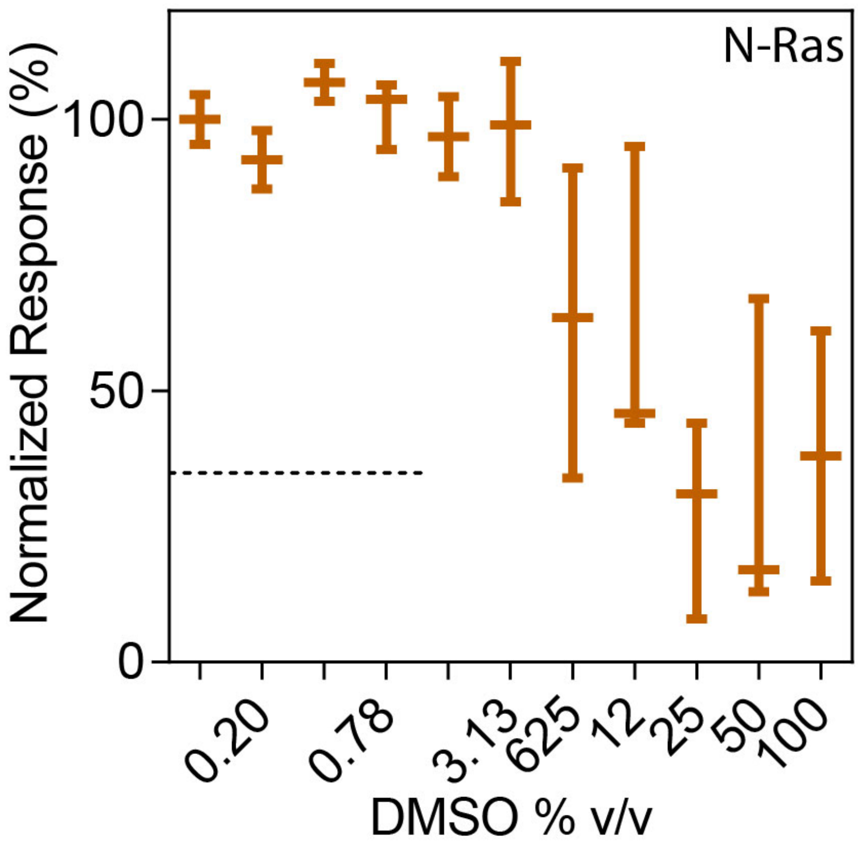

[DMSO] % v/v	0	1.23	1.62	2.14	5.27
**Signal (%)**					
**N-Ras**	100	86 ± 5	89 ± 7	66 ± 16	38 ± 9
**Fyn**	100	94 ± 16	87 ± 7	79 ± 11	56 ± 4

Supplementary Figure S2: DMSO tolerance. A dose-response curve for DMSO tolerance was constructed by adding indicated concentrations of DMSO (%v/v) to the N-Ras palmitoylation reaction. Data shown are average ± SD for 2 independent experiments.

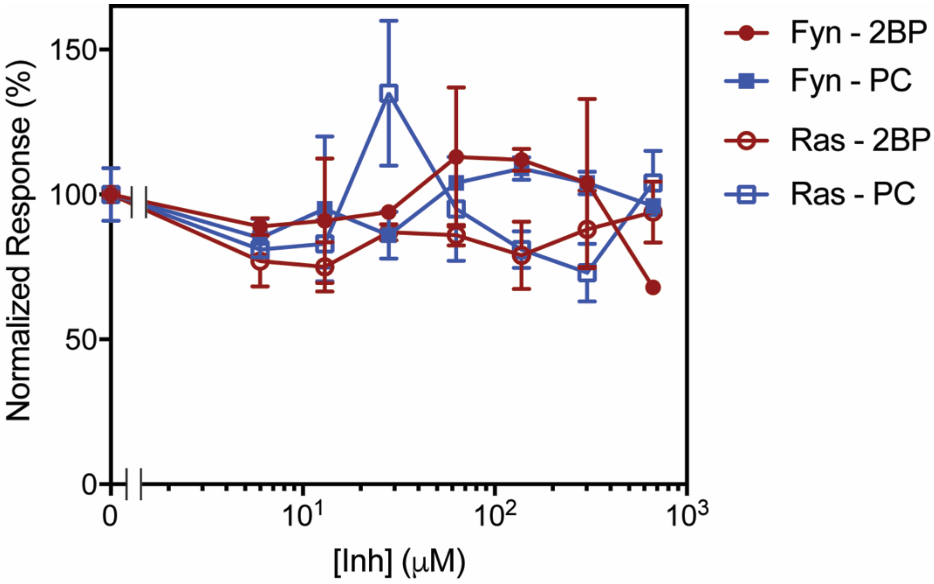

Supplementary Figure S3: Lack of reaction of 2BP and PC with target peptide. Pre-treatment of peptide with known assay inhibitors 2BP and palm-CoA (see Fig 4) had minimal effects on assay response, suggesting that these compounds are inhibiting the palmitoylation enzymes in the membrane preparation rather than reacting with the peptide.

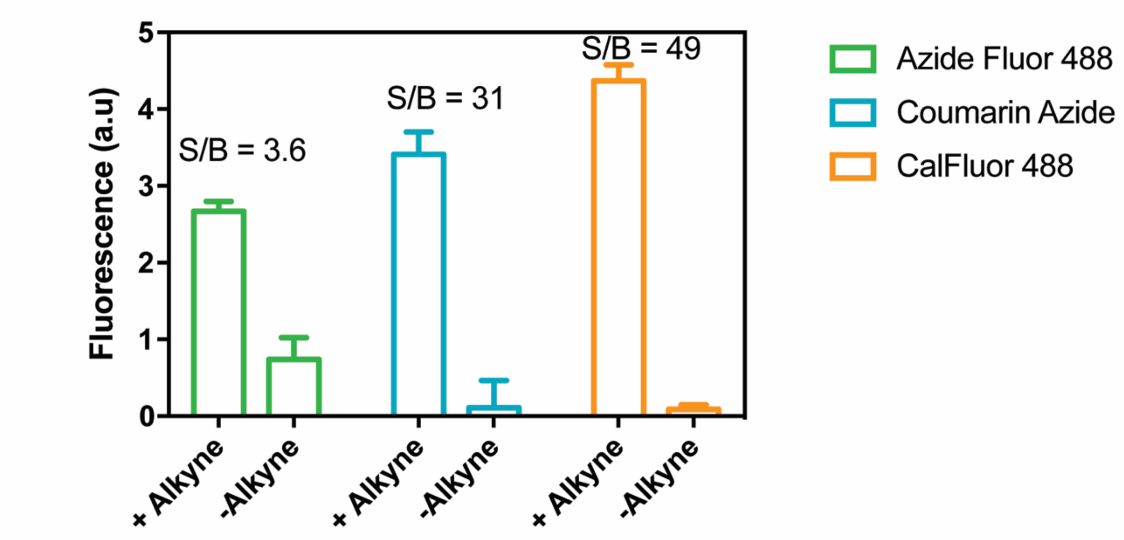

Supplementary Figure S4: Comparison of detection probes. Three different detection probes (AlexaFluor 488 azide; coumarin-azide; and CalFluor 488) were compared in 384-well format by loading streptavidin-coated wells with biotin-alkyne and performing the cyclo-addition reaction with the azido-probes. Blank-subtracted signals from alkyne-containing wells (signal, S) are compared to wells without alkyne (background, B). The fluorogenic coumarin-azide gives an 8-fold increase in S/B, and CalFluor 488 is 1.6-fold more than coumarine-azide. Data shown are average ± SD for triplicates and representative of three independent experiments.
